# The accumulation of Mn and Cu in the morphological parts of* Solidago canadensis* under different soil conditions

**DOI:** 10.7717/peerj.8175

**Published:** 2019-12-10

**Authors:** Aleksandra Bielecka, Elżbieta Królak

**Affiliations:** Siedlce University of Natural Sciences and Humanities, Institute of Biology, Siedlce, Poland

**Keywords:** Heavy metals, Underground parts, Accumulation, Aboveground parts, Phytoremediation

## Abstract

*Solidago canadensis* L. is a drought-tolerant, invasive plant, characterized by a large biomass of underground and aboveground parts. The aim of this study was to assess the accumulation of manganese (Mn) and copper (Cu) in the roots and rhizomes and the stems, leaves, and inflorescence parts in *S. canadensis* from two locations that differed in soil pH, organic carbon, and Mn and Cu concentrations. The concentration of the metals in the samples was determined by the AAS method; the pH was determined by the potentiometric method; and the content of organic carbon was determined using Tiurin’s method. The concentration of Mn and Cu in the roots of *S. candensi*s correlated with the concentrations of the metals in the soil without regard to the soil condition or its organic carbon content. With a low soil pH and organic carbon content, Mn accumulation per 1 ramet in the aboveground parts of *S. canadensis* consisted over 50% of the total Mn content in the plant. In neutral or alkaline soils, the amount of Mn per 1 ramet accumulated in underground parts was over 60%. Regardless of the soil conditions, about 35% of Mn accumulated in rhizomes. Approximately 60% of copper accumulated in the underground parts of *S. candensis* (45% in rhizomes) without regard to the soil reaction or organic carbon content. The ability of the plant to accumulate large amounts of metals disposes *Solidago canadensis* as a candidate for the phytoremediation of soils contaminated with heavy metals.

## Introduction

Manganese (Mn) and copper (Cu) are essential elements for the growth and development of plants (Marschner, 1995). These elements participate in metabolic processes, including oxidation–reduction (redox) reactions in cells, photosynthesis, and the activation of enzymatic reactions (Ducic & Polle, 2005; Millaleo et al., 2010). Plants absorb metals from the soil and the availability of the metals is dependent upon the soil reaction and its organic matter content (Adamczyk-Szabela, Markiewicz & Wolf, 2015; Rosselli, Keller & Boschi, 2003). For example, if the pH of the soil decreases, the absorption of Mn by the plant increases (Watmough, Eimer & Dillon, 2007). However, the influence of soil pH on the bioavailability of Cu for plants is small. Cu, in contrast to Mn, has a strong affinity to bind with organic matter, which decreases the bioavailability of this metal (Reichman, 2002). The distribution of metals in the morphological parts of the plants depends on the species, its growth phase, and on the role microelements play in the metabolic processes. Most heavy metals accumulate in greater quantities in the roots and rhizomes versus the aboveground parts. Metal concentrations localized in particular parts of a plant are the result of their unique absorption and transportation.

A plant that has received substantial attention in Europe and Asia due to its invasive nature is *Solidago canadensis* L. (Dong et al., 2006; Sun et al., 2006; Weber, 2001; Yuan et al., 2013). The species occupies various natural and synanthropic habitats (Guzikowa & Maycock, 1986; Weber, 2000), including mining sites and industrial waste dumps (Skubała, 2011; Vega et al., 2004). *Solidago canadensis* is a perennial plant that reproduces vegetatively and generatively, producing aerial parts annually and reaching a height of up to 2 m. The inflorescence, in the form of a panicle, is located at the top of the stem. Individual clones form dense clusters that produce one or more ramets, depending on the age of the clone (Weber, 2000). The plant produces a large biomass of aboveground parts, estimated at 15.9 Mg dry matter/ha (Ciesielczuk et al., 2016). *S. canadensis* is characterised by an extensive system of underground roots and rhizomes. The ratio of underground parts to the aboveground parts of the plant is in the range of 0.25–0.82 (Wang et al., 2017). The roots propagate from the base of the ramets and reach a minimum depth of 20 cm (Weber, 2000). *S. canadensis* tolerates a range of conditions (Huang, Guo & Chen, 2007; Jin et al., 2004), surviving in soils with a high content of heavy metals, including Zn, Cu, and Pb (Antonijevic et al., 2012; Huang, Guo & Chen, 2007; Yang et al., 2007; Yoon, Cao & Zhou, 2006). Current literature is lacking in information on the content of specific metals in the morphological parts of the plant, including the inflorescences and rhizomes.

Understanding the distribution of metals in individual parts of the plant may lead to the use of the plant for phytoremediation. Species used for this purpose are characterized by rapid growth, an extensive root system, and a resistance to environmental stressors (Jasion et al., 2013; Laghlimi et al., 2015; Yoon, Cao & Zhou, 2006). A plant that is able to absorb contaminants from the soil and translocate them into its aboveground parts can be used as a phytoextractor; however, if contaminants remain in its underground parts, it is regarded as a phytostabilizer (Laghlimi et al., 2015). Despite the widespread distribution of *S. canadensis*, little research has been done on the phytoremediation uses of this plant. The extensive biomass of its underground parts and high biomass of its aboveground parts predisposes *S. canadensis* to being a phytoaccumulator of metals. Research conducted by Fu et al. (2017) indicates that *S. canadensis* can be used for the phytoremediation of soils contaminated with cadmium. The analyses of the Mn and Cu contents in the individual parts of the plant is of interest; the choice of metals for our research was dictated by their bioavailability to plants that are dependent on the specific chemical parameters of the soil.

We hypothesize that the content of Mn and Cu varies among the individual morphological parts of *S. canadensis* and is dependent on the location of the study site. The soil reaction and the content of organic matter were expected to influence the concentrations of the metals in particular parts of the plant. The possibility of using S. canadensis as a phytoremediator of both Mn and Cu is discussed.

## Material & Methods

### Study site

**Figure 1:**
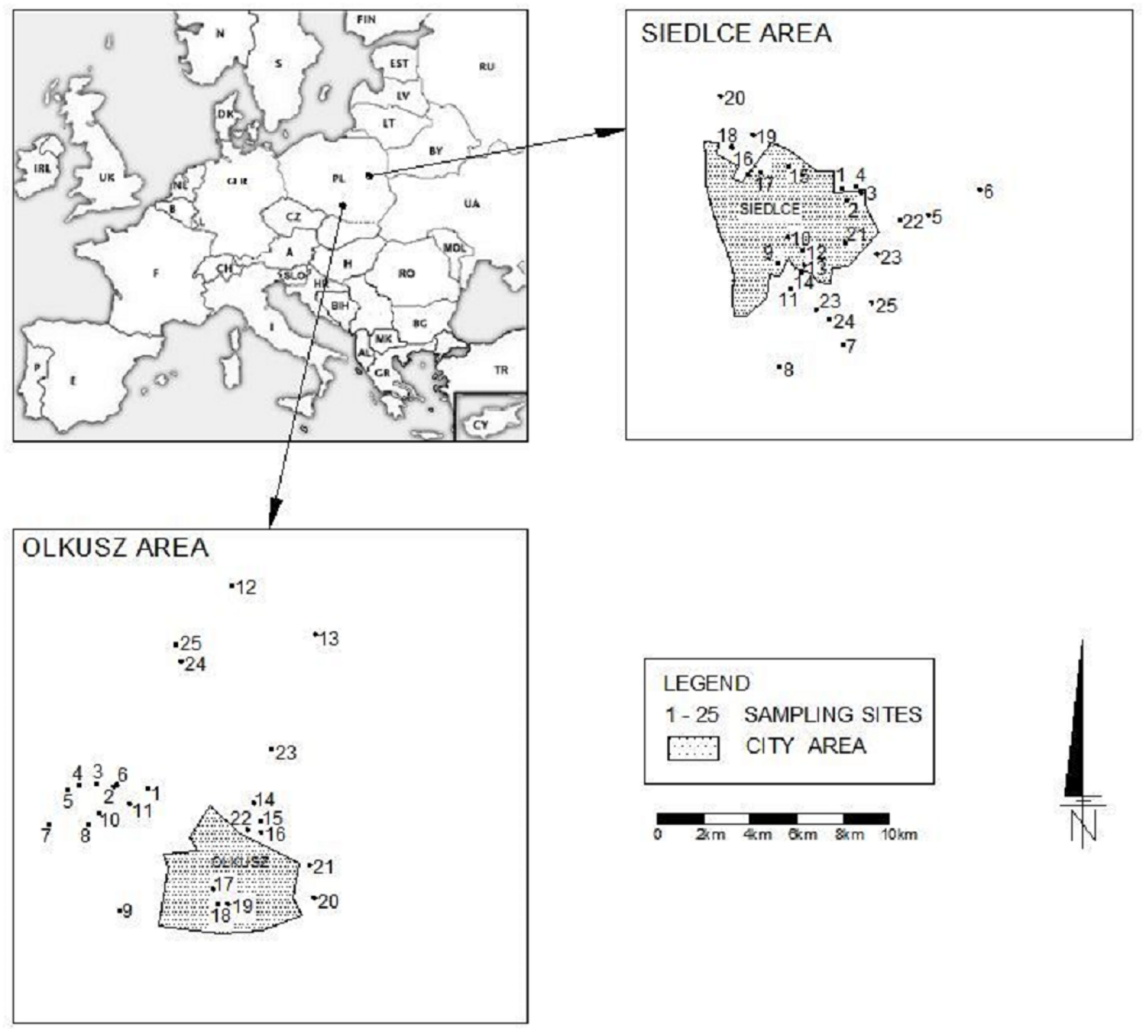
Location of the study area and sampling sites.

The research was carried out in Siedlce (52°10′N, 22°17′E) and Olkusz (50°16′N, 19°33′E) in Poland (Central Europe). The regions are characterized by different types of economic activity; Siedlce is located in an agricultural region in eastern Poland, while Olkusz is located in an industrial region in southern Poland (Fig. 1). Soils in the Siedlce region are characterized by naturally-occurring heavy metals (Siebielec, 2017) and there are high concentrations of heavy metals connected with the mining industry in Olkusz (Kapusta, Szarek-Łukaszewska & Vogt, 2015).

### Sample collection

**Plant**. Twenty-five sites were selected in each location and were characterised by the presence of *S. canadensis* over an area of at least 500 m^2^. Three representative 3 × 3 m plots were designated at each site and the number of ramets was counted. A 40 × 40 cm frame was used to cut a plant patch with a depth of up to 20 cm. The aboveground and underground plant parts were separated. The isolated ramets were counted and then twenty ramets were selected from each site for further analysis. The isolated underground parts of the plants were packed into bags while in the field and subsequently transported to the laboratory.

**Soil.** Four soil subsamples were collected from each plot using an Egner’s sampling stick and these were pooled into a composite sample. Samples collected in the field from each plot were secured in plastic bags, described and transported to the laboratory.

### Laboratory analysis

The roots, rhizomes, stems, leaves and inflorescences were separated. The underground portions were thoroughly rinsed under running water and again in distilled water. The isolated parts were dried at 60 °C to a constant weight. The dried material was weighed and homogenized. The soil samples were air dried in the laboratory, sifted through a sieve with a mesh diameter of 2 mm, and subjected to further analysis. The representative soil subsamples were homogenized using an agate mortar.

Plant and soil samples were initially mineralised in a muffle furnace at a temperature of 420 °C for 6 h and then mineralized in a mixture of HNO_3_ (68%) and H_2_O_2_ (30%) in a microwave (3:2, v/v) (Kabała & Karczewska, 2017).

The soil reaction in 1 M KCl was measured with pH-meter CP-215 (Elmetron) and the organic carbon (OC) content was determined by Tiurin’s method using potassium dichromate as the oxidizer of the organic carbon (Kabała & Karczewska, 2017).

The analyses of the Mn and Cu contents in the mineralised samples were performed by the atomic spectrometric method (manufactured by Carl Zeiss Jena) using an acetylene-air flame. Standard solutions with a concentration ranging from 0.2 to 3.0 µg cm^−3^ for Cu and 0.5 to 5.0 µg cm^−3^ for Mn were used to determine the individual metals. Polish Virginia Tobacco Leaves (INCP–PVTL–6), prepared and certified by the Institute of Nuclear Chemistry and Technology (Warsaw, Poland), were used as a reference material in this study. The contents of Mn and Cu in the reference material were 136.5 mg/kg and 5.12 mg/kg, respectively and our measurements of the same material read 139.9 ± 4.90 mg Mn/kg and 4.97 ± 0.28 mg Cu/kg. Redistilled water of 5 µS cm^−1^conductivity was used in this analysis.

### Calculation and statistical analysis

The dry matter of roots, rhizomes, stems, leaves, and inflorescences per 1 ramet was determined. The accumulation of manganese and copper in the underground and aboveground parts of *S. canadensis* per 1 ramet was calculated. The calculations were based on the concentrations of the chemical elements in 1 kg of dry matter (DM) of the examined morphological parts of the plant and the individual tissues of the plant that make up 1 ramet. The normality of the data was tested using the Shapiro–Wilk test. When the data were not normally distributed we used nonparametric tests. The Mann–Whitney test was used to compare the values of the measured parameters in the samples collected from Siedlce and Olkusz. The significance of the differences in the metal contents in the various morphological parts of the plant and their corresponding soil samples was calculated using the Kruskal-Wallis and Dunn’s test. Spearman’s correlation coefficients were used as a measure of the strength of the relationships between soil pH, organic carbon content, and the metal content in the morphological parts of *S. canadensis*. In order to find a general relationship between the Mn and Cu concentrations in the morphological parts of the plant and the corresponding soil properties, the Principal Components Analyses (PCA) was conducted. The location (in the Siedlce or Olkusz area) was omitted as a variable. In order to compare the biomass of the individual plant parts per 1 ramet (normally distributed data), the *t*-test was used. The statistical analyses were performed using STATISTICA 12 software (StatSoft Inc., 2013, Tulsa, OK, USA). An *α* value of 0.05 was used as the criterion of significance.

## Results

### Soil properties

The locations selected for the study differed in soil acidity, the content of OC, and in their concentrations of Mn and Cu. The soil samples collected in the Siedlce area had a higher acidity compared to the samples collected in the Olkusz area. More than 75% of the soil samples collected in Siedlce were acidic or strongly acidic (pH < 5.5), while only 8% of the samples from Olkusz had a pH below 5.5. The soil samples collected in Olkusz were richer in organic carbon compared to the samples collected in Siedlce. The contents of Mn and Cu were about three times higher and two times higher, respectively, in Olkusz than Siedlce (Table 1).

### Concentrations of metals in the plant

**Figure 2:**
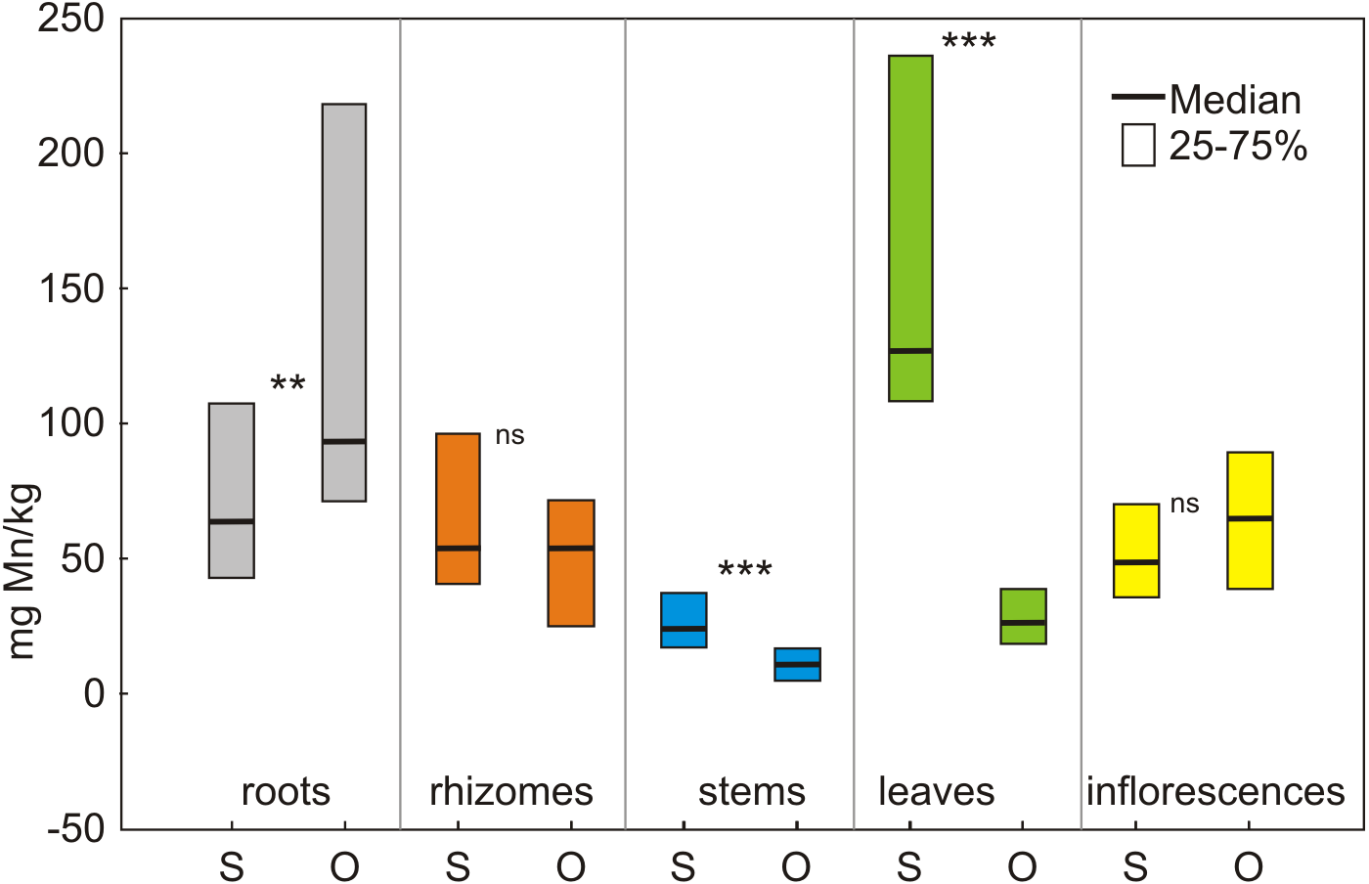
The content of manganese in morphological parts of *S. canadensis* at the sites in Siedlce and Olkusz. S, Siedlce; O, Olkusz; **p < 0.01; ***p < 0.001; ns, not significant.

**Manganese.** The concentration of Mn in specific morphological parts of *S. canadensis* was lower than its concentration in the soil. Only leaves from Siedlce showed a similar concentration of Mn in the soil (Fig. 2; Table 1). Significant differences (*p* < 0.001) were noted between the Mn concentration in specific areas of each location (values of Kruskal-Wallis test: H_4,125_ = 49.50 in Siedlce, H_4.125_ = 87.02 in Olkusz (Table 2)).

The concentration of Mn per 1 kg DW in particular parts of the plant in Siedlce decreased from leaves to roots to rhizomes to inflorescences and finally to the stems. In Olkusz, the concentration decreased from the roots to inflorescences to rhizomes to leaves and finally to the stems (Fig. 2, Table 2).

The comparison of the same morphological parts in both locations revealed that only the concentration of Mn found in the roots from Siedlce was lower than in Olkusz. Mn concentrations were higher or did not significantly differ in other parts between the two locations (Fig. 2).

**Figure 3:**
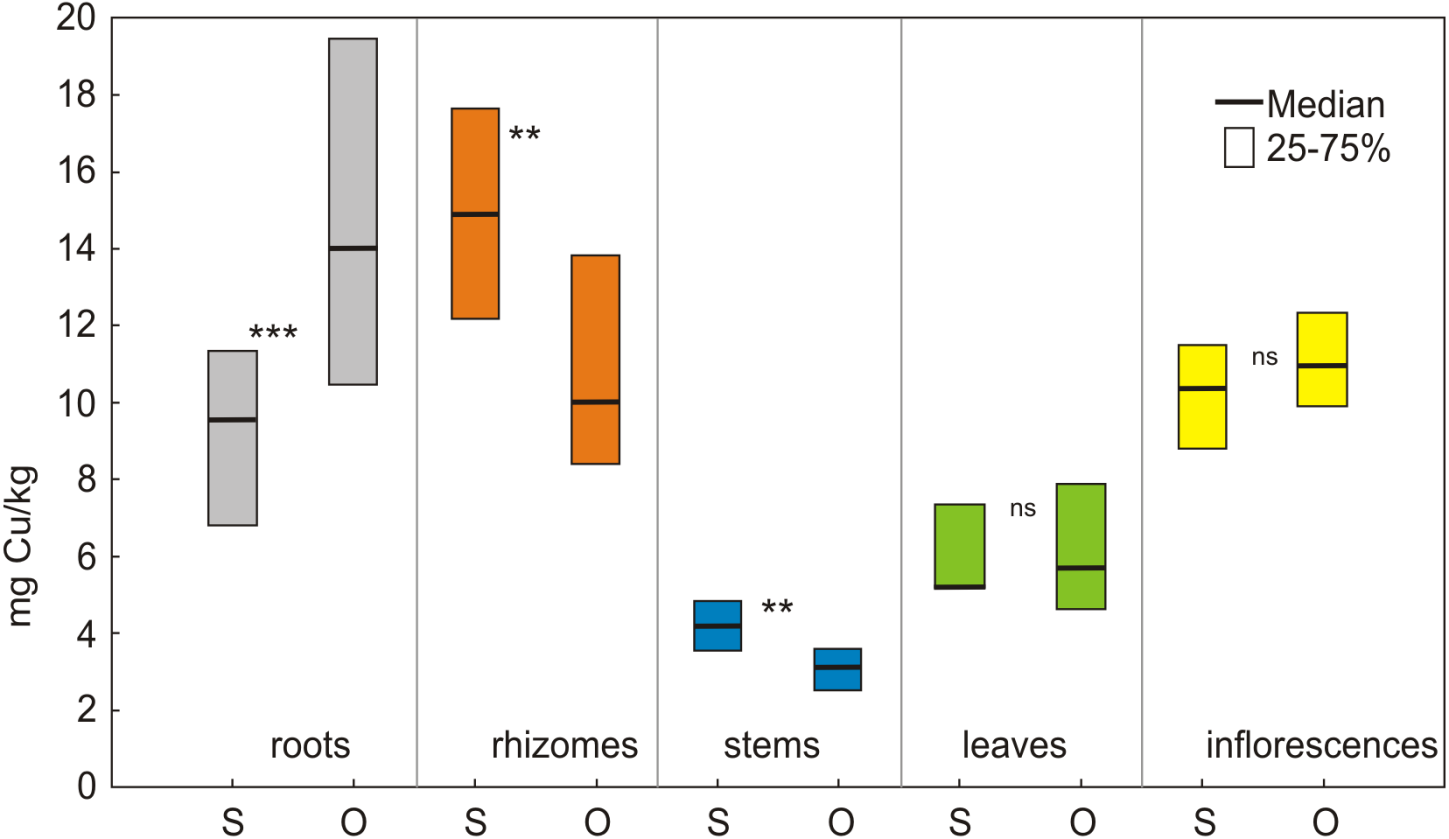
The content of copper in morphological parts of *S. canadensis* at the sites in Siedlce and Olkusz. S, Siedlce; O, Olkusz; **p < 0.01; ***p < 0.001; ns, not significant.

**Copper**. The concentration of Cu in the roots and rhizomes as well as in the inflorescences at both locations was higher compared to the concentration of this element in the soil (Fig. 3; Table 1). Significant differences (*p* < 0.001) were noted between the Cu concentration of metals in particular parts in each location (values of Kruskal-Wallis test: H_4,125_ = 82.10 in Siedlce, H_4.125_ = 86.54 in Olkusz) (Table 2). The concentration of Cu in the morphological parts of the plant in Siedlce decreased from rhizomes to inflorescences to roots to leaves and finally to stems. In Olkusz the concentrations varied from roots to inflorescences to rhizomes to leaves and finally to stems (Fig. 3, Table 2). The Cu concentration was higher in the roots and lower in rhizomes and stems in Olkusz versus Siedlce. The concentration of copper in the leaves and inflorescences was similar at both locations (Fig. 3).

**Table 1:** Soil reaction, the content of OC, Mn and Cu in soil samples collected in Siedlce and Olkusz. M-W, Mann-Whitney test; *N* = 50, *p* < 0.001.

Parameter	Unit	Siedlce		Olkusz		M-W test
		Mean ± SD	Median (Range)	Mean ± SD	Median (Range)	Z
Reaction	pH		4.84 (3.88–7.51)		6.53 (5.00–7.75)	4.49
OC	%	1.66 ± 0.47	1.54 (1.03–2.64)	2.69 ± 1.64	2.19 (1.28–9.70)	3.90
Mn	mg/kg	132.4 ± 71.2	127.3 (13.01–245.6)	406.3 ± 152.4	422.1 (111.0–626.7)	5.22
Cu	mg/kg	6.18 ± 2.86	5.71 (1.37–12.74)	10.32 ± 3.94	10.58 (5.16–23.03)	3.82

**Table 2:** Significance of differences in particular study locations in the content of Mn and Cu in the parts of *S. canadensis* by the Dunn’s multiple range test.

Metal	Parts	Siedlce				Olkusz			
		Rh	St	L	I	Rh	St	L	I
Mn	R		^***^			^**^	^***^		^***^
	Rh		^***^	^*^			^***^		
	St			^***^				^***^	^**^
	L				^***^				
Cu	R	^**^	^***^				^***^	^***^	
	Rh		^***^	^***^			^***^	^***^	
	St				^***^				^***^
	L				^***^				^**^

**Notes.**

R roots Rh rhizomes St stems L leaves I inflorescences

** *p* < 0.01

*** *p* < 0.001

### Interactions between soil properties and the content of Mn and Cu in morphological parts of *Solidago canadensis*

The concentration of Mn in the aboveground parts of the plants was found to increase with a decrease in the soil pH in both study locations. However, an increase in the organic carbon content reduced the concentration of manganese in all parts of the plant (Table 3). In Siedlce, the organic carbon increased the concentration of Cu in the roots of *S. canadensis*. The soil’s pH had no effect on the content of Cu in the tissues of *S. canadensis* in either location. The concentration of Mn and Cu in the soil was correlated with the concentration of these metals in the roots of the plant in both locations.

**Table 3:** Values of Spearman’s correlation coefficients illustrating the relationship between soil properties and metal content in individual morphological parts of *S. canadensis* in Siedlce and Olkusz.

Metal	Parameter	Sites	Roots	Rhizomes	Steams	Leaves	Inflorescences
Mn	pH	S	−0.64^***^	−0.84^***^	−0.79^***^	−0.81^***^	−0.82^***^
		O	0.24	−0.06	−0.25	−0.14	−0.26
	OC	S	−0.37	−0.39^*^	−0.59^**^	−0.48^*^	−0.46^*^
		O	−0.42^*^	−0.21	−0.56^**^	−0.29	−0.51^**^
	Mn in soil	S	0.50^*^	0.17	0.21	0.11	0.06
		O	0.43^*^	0.40^*^	0.44^*^	0.48^*^	0.43^*^
Cu	pH	S	0.32	−0.04	−0.11	−0.24	−0.12
		O	0.26	−0.04	−0.17	−0.14	−0.17
	OC	S	0.57^**^	0.27	0.17	0.19	0.05
		O	0.05	−0.26	−0.38	0.13	0.02
	Cu in soil	S	0.49^*^	0.28	0.43^*^	0.11	0.24
		O	0.46^*^	−0.24	−0.05	0.17	0.02

**Notes.**

S Siedlce O Olkusz

*N* = 50

* *p* < 0.05

** *p* < 0.01

*** *p* < 0.001

**Figure 4:**
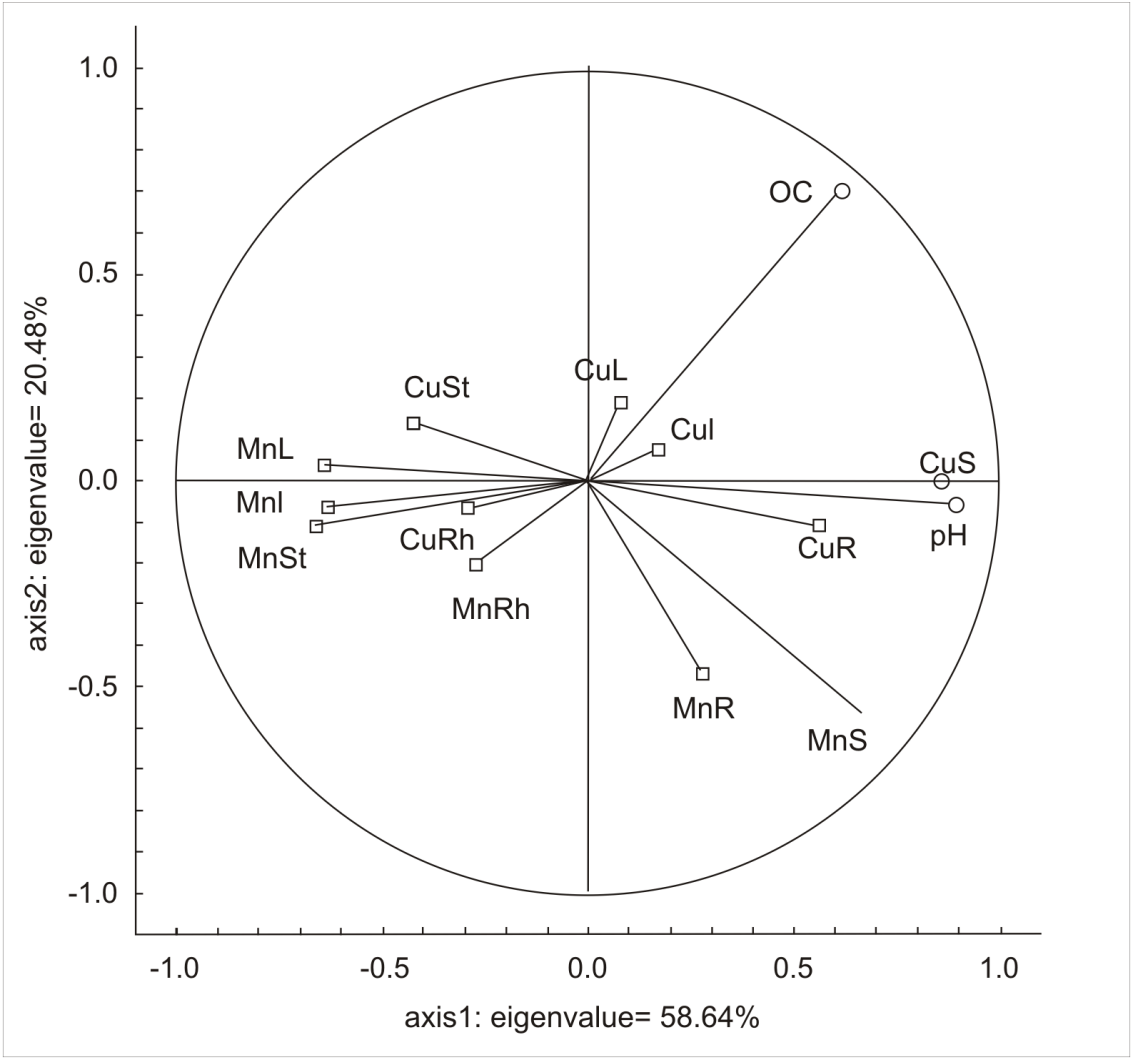
PCA results presenting the relationships between soil pH, OC, Mn and Cu content in soil and the content of Mn and Cu in parts of *S. canadensis*. CuS, Cu content in soil; CuR, Cu content in roots; CuRh, Cu content in rhizomes; CuSt, Cu content in stems; CuL, Cu content in leaves; CuI, Cu content in inflorescences; MnS, Mn content in soil; MnR, Mn content in roots; MnRh, Mn content in rhizomes; MnSt, Mn content in stems; MnL, Mn content in leaves; MnI, Mn content in inflorescences.

Interrelations between the tested soil properties and the concentration of metals in the underground and aboveground parts of the plant, regardless of the location, are illustrated in Fig. 4. PCA1 was strongly associated with the soil pH and Mn content in soil. The higher pH of the soil was correlated with a lower Mn concentration in the aboveground parts of the plants (leaves, stems, inflorescences). This axis defined approximately 60% of the total amount of variation. PCA also corroborated correlations between the soil pH and the OC content (R_s_ = 0.55), soil pH and Mn concentrations (R_s_ = 0.45), soil pH and Cu concentrations (R_s_ = 0.75), and OC and Cu concentrations in the soil (R_s_ = 0.60).

### The percentage contribution of the morphological parts of *S. canadensis* in the accumulation of Mn and Cu per 1 ramet

**Figure 5:**
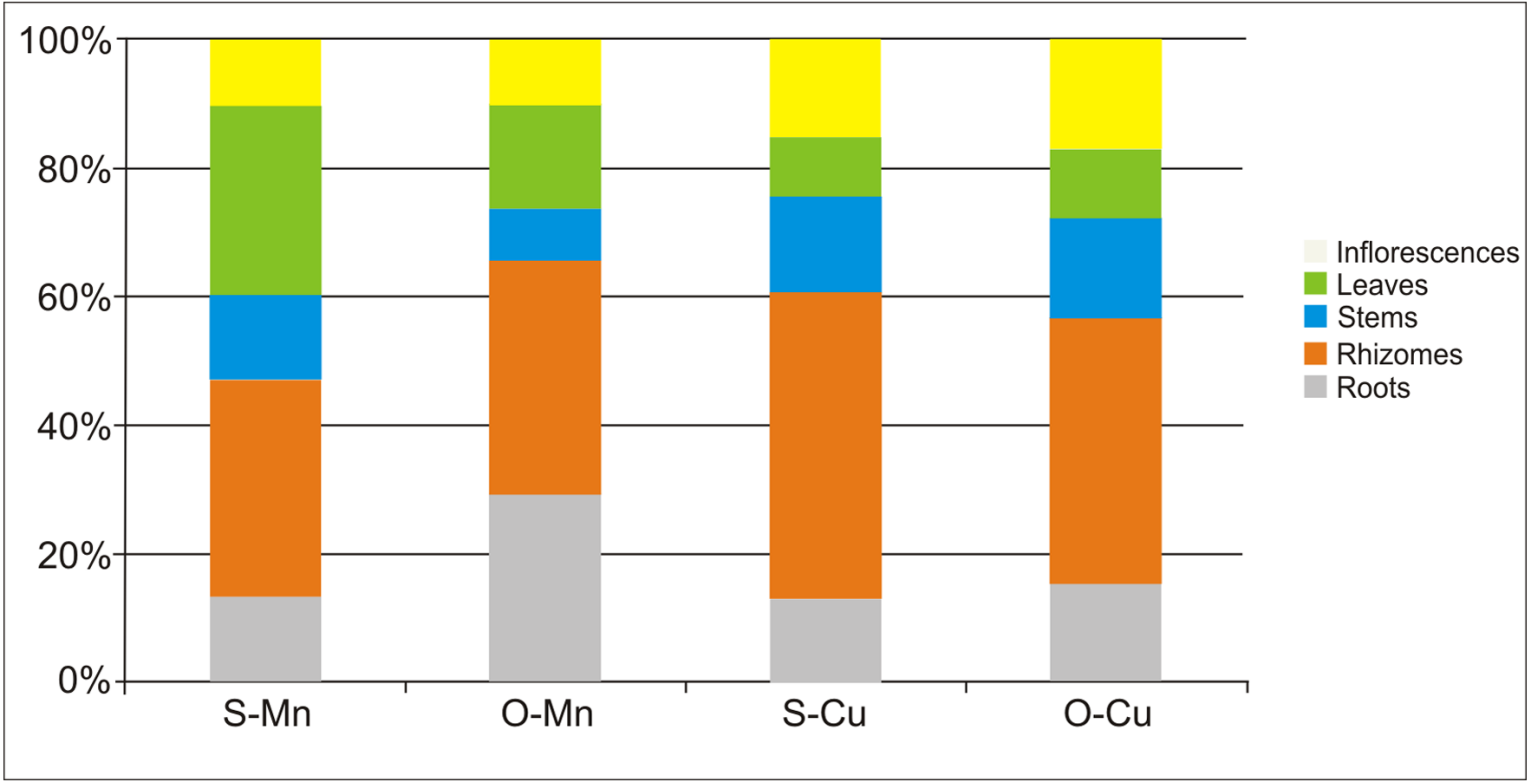
Average percentage contribution of different morphological parts of *S. canadensis* calculated per 1 ramet in the accumulation of Mn and Cu in Siedlce and Olkusz.

The plants from Siedlce had a more developed root system but were less leafy than the plants from Olkusz (Table 4). The contents of Mn and Cu in the underground and aboveground parts of *S. canadensis* was calculated per 1 ramet based on the dry matter calculated for the morphological plant parts per 1 ramet (Table 4) and the concentration of metals in 1 kg DM of the analysed parts. The average percentage contribution of the morphological parts of *Solidago* in the accumulation of the analysed metals is presented in Fig. 5.

The roots and rhizomes collected in Siedlce accumulated approximately 47% of Mn and those from Olkusz accumulated approximately 66%. The contribution of the underground parts of the plant in the accumulation of copper was over 50% in either location. Rhizomes contributed to an accumulation of about 35% of manganese and about 45% of copper.

## Discussion

The studied locations differed from each other in soil reaction, organic carbon content, and in the content of Mn and Cu in the soil. Soils in the agricultural region of Siedlce were characterized by a higher acidity and a lower concentration of metals when compared to those of Olkusz. The concentration of metals in the individual parts of the goldenrod differed between the two locations. In both locations, the smallest Mn and Cu concentrations were noted in stems. The highest Mn and Cu concentrations were found in the roots in Olkusz, while Mn was the highest in leaves and Cu was the highest in the rhizomes taken from Siedlce. Jasion et al. (2013), Nadgórska-Socha, Kandziora-Ciupa & Ciepał(2015), and Yoon, Cao & Zhou (2006) emphasized that the accumulation of metals in plant tissues is determined by the type of metal and the corresponding soil properties (Adamczyk-Szabela, Markiewicz & Wolf, 2015). According to Watmough, Eimer & Dillon (2007), the availability of Mn for plants increases with a decrease of pH in soil. Soluble forms of Mn, which are dominant in acidic soils with pH <5.5, are particularly available for plants (Ducic & Polle, 2005). In our study most soil samples in Siedlce were characterized by low pH values (below 5.5), which could have contributed to greater manganese mobility. This is indicated by significant negative values of correlation coefficients between the soil pH and the metal content in individual morphological parts of *S. canadensis.* This was confirmed by the results of PCA analysis indicating an opposite relationship between the soil pH and Mn content in the aboveground parts of the plant. The effect of pH on Cu uptake by plants is negligible (Takáč et al., 2009), which was also confirmed by the results of our research.

**Table 4:** Average dry matter ± SD [g] of morphological parts calculated per 1 ramet; the values of *t*-test (df = 48), *N* = 50 (Bielecka & Królak, 2019).

Part of the plant	Siedlce	Olkusz	*t*-test
Inflorescences	2.31 ± 1.00	2.49 ± 0.69	*t* = 0.72, *p* = 0.47
Leaves	2.37 ± 0.90	2.90 ± 0.91	*t* = 2.07, *p* = 0.04
Stems	5.70 ± 2.57	6.58 ± 1.90	*t* = 1.38, *p* = 0.17
Rhizomes	5.03 ± 1.82	4.74 ± 2.65	*t* = 0.44, *p* = 0.66
Roots	2.25 ± 0.61	1.59 ± 0.74	*t* = 3.43, *p* = 0.001

Apart from the soil pH, an important factor determining the mobility and phytoavailability of metals in the soil is the organic carbon content, which is a factor that limits the uptake of metals by plants (Gupta & Sinha, 2007; Fijalkowski et al., 2012; Reichman, 2002). The results of our research showed that a high content of organic carbon in the soil limits the uptake of Mn by individual parts of *Solidago*. However, no effect of OC on the Cu concentration in the aboveground parts of the plant was found. It was also noticed that in both locations Cu predominantly accumulated in the underground parts of *S. canadensis*.

The concentration of Mn and Cu in inflorescences did not differ significantly between Siedlce and Olkusz. De Lima et al. (2015) noted that there is a high demand for these elements during the flowering period of the plant. Similarly, Gaweda (2009) emphasized the high accumulation of Cu by inflorescences. In our research, the lowest concentrations of metals in both locations were found in the stems of *S*. *canadensis*. The results indicate that the *S. canadensis* stems are not Mn and Cu accumulators and only serve as transporters of the metals to the leaves and inflorescences. Similar dependencies for other plant species were obtained for *Rumex* sp. by Gaweda (2009), for lavender by Zheljazkov & Nielsen (1996), and for tobacco by Bozihova & Bozhinov (2006).

Metals accumulate in plants as the result of many factors including the bioavailability of metals, organic carbon content, soil pH, their interactions with other elements, and the biomass of the particular parts of plants (Fijalkowski et al., 2012; Siebielec, Stuczyński & Korzeniowska-Puculek, 2006). The ratio of the underground to aboveground parts of *S. canadensis* was 0.81 in Siedlce and 0.54 in Olkusz on average and this ratio was within the range of 0.25–0.82 reported by Wang et al. (2017). The extensive system of underground parts of *S. canadensis* may play an important role in the accumulation of heavy metals in these morphological parts. In addition, an extensive system of rhizomes can increase soil stabilization and reduce its erosion, which is important in cases where the plant colonizes mining sites and industrial waste dumps (Skubała, 2011; Vega et al., 2004).

Plants that are characterized by a high biomass are tolerant to contaminated environments and are capable of accumulating metals; the plants are often used as phytoremediators for polluted soils. They are represented by *Miscanthus giganteus* (Dražić et al., 2017) and *Helianthus tuberosus* L., among others (Jablonski et al., 2015). Our research results indicate that, similar to *Miskant giganteus* (Dražić et al., 2017), *S. canadensis* accumulates more Cu in its underground than aboveground parts, while the aforementioned plants accumulate more Mn in the aboveground parts than the underground ones.

Laghlimi et al. (2015) emphasized the need to identifying more species with remediative abilities. Fu et al. (2017) indicated that *S. canadensis* has potential for the phytoremediation of soils that have been contaminated with cadmium. Our research showed that *S. canadensis* may also be a Cu-phytostabilizer and Mn-phytoextractor. Phytoremediation processes are most effective in soils contaminated marginally to moderately (Laghlimi et al., 2015). The mean content of Mn and Cu in the soils of both locations corresponded to the levels accepted as being natural for Polish soil (Kabata-Pendias et al., 1995). The results of the present research revealed that Cu accumulated in the underground plant parts while manganese accumulated in the leaves. This reflects the findings of Antonijevic et al. (2012) who conducted their study in a Serbian region heavily contaminated with copper known as the Copper Mining and Smelting Complex.

## Conclusions

Individual morphological parts of *Solidago canadensis* show varying concentrations of Mn and Cu. The plants in low pH soil had the highest concentrations of Mn in the leaves, whereas in neutral or alkaline soil, the highest concentrations of Mn were found in the roots. A higher content of organic carbon reduced the Mn accumulation in the aboveground parts of *S. canadensis*. The underground parts of the plant had a higher Cu concentration than the aboveground parts. The soil conditions did not have a significant influence on the Cu content in the individual parts of *S. canadensis*.

The extensive underground system of *Solidago* and the high accumulation of Cu in the roots and rhizomes irrespective of soil conditions predisposes this species to act as a Cu-phytostabilizer. It may also be a Mn-phytostabilizer in soils with a high organic carbon content and neutral pH.

##  Supplemental Information

10.7717/peerj.8175/supp-1 Tables S1 and S2 The content of manganese and copper in morphological parts of *S. canadensis* at the sites in Siedlce and Olkusz

10.7717/peerj.8175/supp-2 Figure S3 Average percentage contribution of different morphological parts of *Solidago canadensis* calculated per 1 ramet in the accumulation of Mn and Cu in Siedlce and Olkusz

10.7717/peerj.8175/supp-3 Table S1 Soil reaction, the content of OC, Mn and Cu in soil samples collected in Siedlce and Olkusz

10.7717/peerj.8175/supp-4 Table S2 Dry matter (DM) [g] of morphological parts calculated per 1 ramet

## Additional Information and Declarations

### Data Availability

The following information was supplied regarding data availability: The raw measurements are available in the Supplemental Files.
